# Exosome-Derived microRNA: Implications in Melanoma Progression, Diagnosis and Treatment

**DOI:** 10.3390/cancers15010080

**Published:** 2022-12-23

**Authors:** Qiang Ye, Zi Li, Yang Li, Yirong Li, Yan Zhang, Runlin Gui, Yue Cui, Qi Zhang, Lu Qian, Yuyan Xiong, Yi Yu

**Affiliations:** 1 Xi’an Key Laboratory of Cardiovascular and Cerebrovascular Diseases, Xi’an No. 3 Hospital, The Affiliated Hospital of Northwest University, Faculty of Life Sciences and Medicine, Northwest University, Xi’an 710069, China; 2 Key Laboratory of Resource Biology and Biotechnology in Western China, Ministry of Education, School of Medicine, Northwest University, Xi’an 710069, China; 3 Department of Endocrinology, Xi’an No. 3 Hospital, The Affiliated Hospital of Northwest University, Northwest University, Xi’an 710069, China

**Keywords:** microRNA, exosome, melanoma, diagnosis, treatment

## Abstract

**Simple Summary:**

Melanoma is a malignant and invasive cancer with high mortality and poor prognosis, and the incidence is increasing yearly. However, the current therapy options for melanoma are still insufficient and challenging. Recently, exosomes as essential intercellular messengers are receiving great interest for their important roles in melanoma progression, diagnosis, and treatment. Especially, the miRNAs derived from the exosomes are crucial during this process. Here, we summarized the miRNAs derived from melanoma exosomes, and analyzed their function in the progression of melanoma, including invasion and metastasis, microenvironment establishment, angiogenesis, and immune escape. In addition, we also listed the potential miRNAs including exosomal miRNAs as diagnostic markers for melanoma and introduced new therapeutic strategies for melanoma based on miRNAs and exosomes.

**Abstract:**

Melanoma is a malignant and aggressive cancer, and its progression is greatly affected by interactions between melanoma cells and their surroundings. Exploration on mechanism of melanoma and improved diagnostic and therapeutic strategies are becoming increasingly important. Unlike extracellular messengers that mainly work on targeted cells through corresponding receptors, exosomes are essential intercellular messengers that deliver biologically active substances such as nucleic acids and proteins to target cells for cell–cell communication. Of them, microRNAs (miRNAs) are common and important exosomal components that can regulate the expression of a wide range of target genes. Accordingly, exosome-derived miRNAs play a significant role in melanoma progression, including invasion and metastasis, microenvironment establishment, angiogenesis, and immune escape. MiRNA signatures of exosomes are specific in melanoma patients compared to healthy controls, thus circulating miRNAs, especially exosomal miRNAs, become potential diagnostic markers and therapeutic targets for melanoma. This review aims to summarize recent studies on the role of exosomal miRNAs in melanoma as well as ongoing efforts in melanoma treatment.

## 1. Introduction

Melanoma is a type of malignant skin cancer derived from melanocytes. According to the epidemiological assessment of global cancer data, 325,000 new melanoma cases and 57,000 deaths from melanoma occurred in 2020. If the incidence rate remains stable, the global melanoma burden is estimated to rise to 510,000 new cases and 96,000 deaths by 2040 [1]. Oxidative stress and DNA damage caused by ultraviolet are the leading causes of melanoma. High mortality and poor prognosis make melanoma the most common skin tumor-related cause of death, due to its strong aggressive and metastatic ability. Until now, the treatment options for melanoma, such as surgical resection, radio-chemotherapy, and even targeted therapy and immunotherapy are still insufficient and limited. Accordingly, an in-depth understanding of the mechanisms for melanoma development is necessary and critical for its prevention, diagnosis, and treatment.

Recently, exosomes are gaining much interest due to their crucial functions in cell-to-cell communication and their exceptional qualities, including high stability, low cytotoxicity, low immunogenicity, and high membrane permeability. Exosomes are membrane-bound vesicles, and abundantly exist in different body fluids. Recent studies document that tumor cells release exosomes frequently, and these exosomes are crucial for several early and late events associated with tumor development and metastasis, which largely depends on the transport of different biomolecules, including proteins, RNAs, and lipids from tumor cells to their surroundings. For example, the metastatic breast cancer cell line MDA-MB-231 was reported to damage the tight junction of the endothelial monolayer, leading to increased migration and metastasis, which mainly depends on the exosomal miR-105 derived from cancer cells to endothelial cells [2]. In addition, the clinical application of exosomes as therapeutic carriers has aroused great interest [3], since their small size and membrane composition allow them to cross major biofilms, even the blood–brain barrier. Among the various exosomal carry-ons, miRNAs have far more significant effects, which have been well demonstrated to be involved in many biological activities, such as cell proliferation, differentiation, migration, disease initiation, and progression. For instance, miR-21 that can be detected in exosomes derived from various cancers such as hepatocellular carcinoma [4] and colon adenocarcinoma [5], was revealed to promote hepatocellular carcinoma growth through Tet methylcytosine dioxygenases/PTEN pseudogene 1/Phosphatase and tensin homolog (TETs/PTENp1/PTEN) pathway [4], and facilitate colon adenocarcinoma proliferation and invasion by targeting programmed cell death 4 (PDCD4) [5]. Therefore, exosome-derived miRNAs are extensively studied and developed for diagnosis and treatment in a variety of cancer models, including melanoma.

In melanoma, numerous exosomal miRNAs have been found and studied from clinical and experimental research, and great efforts have been made to reveal the functions and molecular mechanisms of these miRNAs in melanoma. Here, we mainly focus on the miRNAs derived from melanoma exosomes, summarize and analyze their effects on the progression of melanoma from different perspectives, including invasion, metastasis, angiogenesis, microenvironment, and immune escape, and also give a comprehensive and updated overview of their potential as makers for melanoma diagnosis as well as the advantages and limits of their application in melanoma treatment. We believe that this knowledge will guide us to further understand the roles of miRNAs in melanoma, and provide some suggestions and references for the future clinical development of melanoma treatment.

## 2. Biological Properties and Biogenesis of Exosomes

**Figure 1:**
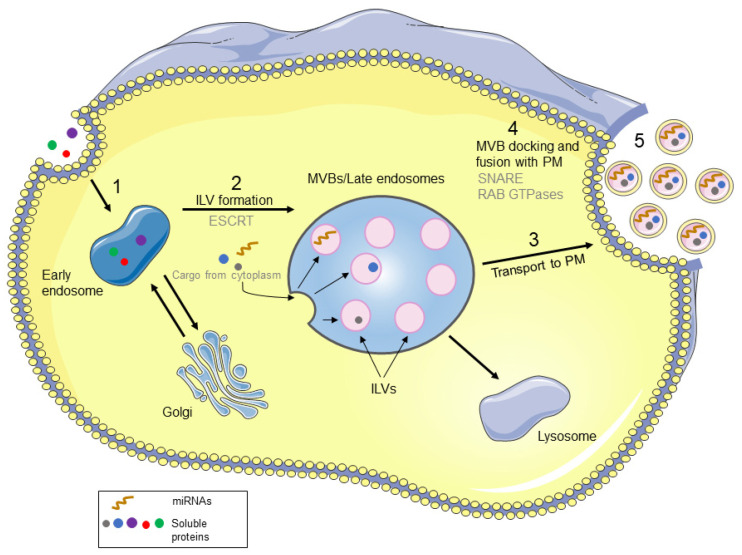
Biogenesis of exosomes. (1) Cargoes are internalized to form early endosomes. (2) Cytoplasmic compartments are sorted into ILVs to form MVBs. (3) MVBs containing exosome cargo are transported to the plasma membrane through the cytoskeleton and microtubule network. (4) MVBs are docked with the plasma membrane through SNARE and Rab GTPases. (5) ILVs are released as exosomes by exocytosis. ESCRT, endosomal sorting complex required for transport; MVBs, multivesicular bodies; ILVs, intraluminal vesicles; PM, Plasma membrane; SNARE, soluble NSF attachment protein receptors.

Exosomes are small, cup-shaped, and membrane-bound vesicles with diameters from 30 to 150 nm, that were first identified in 1981 [6]. Exosomes can be secreted by variety of cells, including normal cells and tumor cells [7], and abundantly exist in almost all body fluids, such as blood, saliva, urine, and so on [8]. So far, the specific mechanisms for exosome biogenesis and secretion have not been fully elucidated, but it is generally accepted that multivesicular bodies (MVBs) are involved in this process, as shown in Figure 1. The plasma membrane first invaginates through endocytosis to form early endosomes, including cell surface proteins and soluble proteins related to the extracellular environment. Some of the early endosomes are recycled by the Golgi apparatus [9], but for the majority, their membrane further invaginates and wraps up proteins and nuclear acids in the cytoplasm to form MVBs which contain mickle intraluminal vesicles (ILVs), the precursors of exosomes [10]. During this process, the Golgi complex fuses with the membrane of endosomes or MVBs, contributing to the mixed membrane components of exosomes that contain molecules from the Golgi such as MHC class II or the cell surface like growth factor receptors [11,12]. The sorting of transmembrane substances into ILVs largely depends on the endosomal sorting complex required for transport (ESCRT) sorting system [13]. In addition, an ESCRT-independent route was also reported, which requires sphingosine 1 phosphate (S1P), tetraspanin-enriched microdomains and ceramide [14,15]. Afterward, MVBs will either fuse with lysosomes to be degraded or combine with the plasma membrane to release contained ILVs as exosomes [16]. In the latter process, MVBs are transported to the plasma membrane through the cytoskeleton and microtubule network, and then undergo exocytosis by fusion with the plasma membrane, which is regulated by soluble NSF attachment protein receptors protein (SNARE) and its effectors, such as Rab GTPases [17]. Usually, the formation and secretion of exosomes could be adjusted by some proteins and signaling. For example, overexpression of Rab 27a and Rab 27b increases the production of exosomes in melanoma due to their favor for transportation and secretion of MVBs [18]. P53 was also reported to activate exosome production by regulating the transcription of the STEAP3 metalloreductase (TSAP6) gene [19]. Subsequently, exosomes secreted in the extracellular space can fuse with the target cells by binding to its membrane via receptor-ligand interaction or be taken up by recipient cells through endocytosis. The proteins, all kinds of DNAs, and RNAs derived from the donor cells carried by exosomes will be transported accordingly to receipt cells, which leads to the communication between cells and adjustment of the latter.

## 3. Exosome-Derived microRNAs

### 3.1. Sorting of miRNAs into Exosomes

As crucial intercellular messengers, exosomes are involved in the regulation of various physiological or pathological conditions, which is highly dependent on the contents they carry. Of them, miRNAs that are delivered through exosomes and mediate intercellular signal transduction are particularly significant [20,21,22]. MiRNA is a single-stranded RNA composed of approximately 22 nucleotides, which can regulate gene expression after transcription through interaction with RNA. A single miRNA can control multiple mRNA targets. Meanwhile, different miRNAs can also jointly control a single mRNA target, thus forming a complex miRNA–mRNA network.

**Figure 2:**
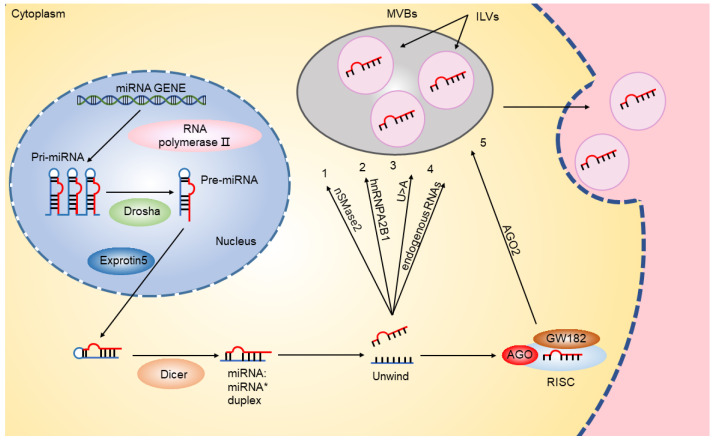
Generation of miRNAs and their sorting mechanisms. In the nucleus, miRNA is first transcribed by polymerase II to produce Pri-miRNA. The ribonuclease type III enzyme Drosha processes Pri-miRNAs to produce hairpin precursors (pre-miRNAs) composed of about 70 nucleotides. Pre-miRNA hairpins are exported to the cytoplasm by EXPORTIN5 and Rnase Ⅲ protein Dicer further works Pre-miRNA into 19–25 nt miRNA double stranded structure. The two less stable strands of the double strand are merged into a multi-protein nuclease complex, the RNA-induced silencing complex (RISC). Mature miRNAs are sorted into exosomes through five mechanisms: (1) Sphingomyelinase 2-dependent pathway. (2) miRNA motifs dependent pathway. (3) 3′-end modification of miRNA-dependent pathway. (4) Endogenous RNA-mediated pathway. (5) miRISC-related pathways. AGO: argonaute RISC catalytic component; GW182, trinucleotide repeat containing adaptor 6A; hnRNPA2B1, heterogeneous nuclear ribonucleoprotein A2/B1; miRISC, miRNA induced silencing complex; MVB, multivesicular body; ILV, intraluminal vesicle.

Given the biogenesis process of exosomes, the composition of exosomal membrane and the contents inside including proteins, RNAs, DNAs, amino acids, and metabolites are usually considered to be similar to that of the host cells. However, numerous studies in different cells found that the miRNA composition in exosomes is different from that in parent cells [23,24], and also varies from cell to cell [25,26], implicating that there might be a mechanism for selecting miRNAs that are preferred to be packaged in the exosomes. Later, researchers further discovered that some miRNAs are more likely to reside in the parent cells, while some are prone to be packaged in exosomes. For exosomes, certain miRNA sequences, such as miR-320 and miR-150, are favored [27]. Currently, the mechanism of sorting miRNAs into exosomes is still not clear, but there are five possible theories, as shown in Figure 2: (1) sphingomyelinase 2-dependent pathway. Sphingomyelinase 2 is the first molecule reported to be associated with miRNA secreting into exosomes. Kosaka et al. [28] demonstrated that sphingomyelinase 2 promotes the secretion of extracellular miRNA. (2) miRNA motifs-dependent pathway. A recent study showed that specific sequences in miRNAs determine whether they are secreted in extra vesicles or retained in cells. And they also defined one of the strongest motifs (CGGGAG) driving miRNA into exosomes through combination of two RNA-binding proteins, Alyref and Fus [29]. Similarly, Villarroya-Beltri et al. [30] found that the GGAG in the 3′-part of the miRNA sequence can be identified by sumoylated heterogeneous nuclear ribonucleoprotein A2/B1 (hnRNPA2B1) and latter lead to packaging miRNAs into exosomes. (3) 3′-end modification of miRNA-dependent pathway. Koppers-Lalic et al. [31] found that miRNAs with the poly (U) in the 3′-end are relatively enriched in exosomes, while the 3′-end adenylated miRNAs mainly exist in cells. (4) Endogenous RNA-mediated pathway. It was demonstrated that the changes of miRNA target levels in the producingcells could mediate miRNA sorting to exosomes by promoting miRNA trafficking from the cytoplasm to MVBs [22]. (5) miRNA-induced silencing complex (miRISC)-related pathway. As known, mature miRNAs can interact with assembly proteins to form a complex called miRISC, consisting of miRNA, miRNA-repressible mRNA, trinucleotide repeat containing adaptor 6A (GW182), and argonaute RISC catalytic component 2 (AGO2). Human AGO2 protein tends to bind to U or A at the 5′-end of miRNA and thus plays a significant role in mediating mRNA-miRNA formation and sorting to exosomes [32]. Guduric-Fuchs et al. [33] and Frank et al. [32] found that AGO2 gene knockout in HEK293T cells causes a prominent reduction in extracellular miRNA releases, such as exosomal miR-142-3p, miR-150 and miR-451. Moreover, it was found that the main components of miRISC were co-located with MVBs [34], thus blocking the transformation of MVBs to lysosomes may lead to the excessive accumulation of miRISC [35]. In conclusion, the five theories above are neither independent nor at odds with each other. The specific sequences or modification present in some miRNAs may guide their incorporation into exosomes, while the abundance and activity of some relevant proteins in different cells may also regulate the categorization of exosomal miRNAs in a manner independent of miRNA sequences.

### 3.2. miRNAs in Melanoma-Derived Exosomes

Like other cancers, melanoma secretes exosomes enriched in diverse small RNA species. Likewise, the miRNA profiles in melanoma-derived exosomes are unique regardless of their cell line of origin. Vignard et al. [36] performed high-throughput sequencing through Affymetrix miRNA 4.0 Arrays and investigated the miRNA contents in melanoma-derived exosomes in different human melanoma cell lines M113, M117, M28, M45, and M6. Based on these, they identified 250 miRNAs enriched in melanoma-derived exosomes that can control gene expression in recipient cells. Of them, 44 miRNAs are the most representative, such as hsa-let-7c-5p, hsa-miR-181a-5p, hsa-miR-3665, and hsa-miR-7704. Furthermore, there was less similarity between the ratio of miRNAs in exosomes and that in their parent cells than between exosomes of different melanoma cell lines. It was demonstrated that 198 miRNAs are downregulated and 206 miRNAs are upregulated in exosomes compared with their donor cells. This preference may be due to a conserved minimal G-rich motif analyzed by MEME, that is miRNAs containing this motif could be specifically sorted into exosomes. Instead, Xiao et al. [37] revealed that the exosomal miRNome largely represents miRNA signatures within their originating cells by analysis of miRNA arrays in human melanoma cell line A375, and only 28 miRNAs upregulated and 5 miRNAs downregulated were observed in exosomes versus parent cells. However, there are differentially expressed miRNAs in A375 exosomes compared to melanocyte exosomes, with 130 miRNAs upregulated and 98 miRNAs downregulated. These miRNAs are linked to the function of cancer, such as hsa-miR-31 and hsa-miR-185 which are related to aggressive features of melanoma, and hsa-miR-34b which is involved in melanoma invasiveness [37]. Notably, the top two upregulated miRNAs, miR-494 and miR-665 in A375 cell-derived exosomes compared with the parent cells are also significantly higher in A375-secreted exosomes than those in melanocyte exosomes. In addition, many studies also reported other highly expressed miRNAs in exosomes derived from different melanoma cells, such as miR-155-5p in B16F10 [38], hsa-miR-100-5p, hsa-miR-99b-5p, hsa-miR-221-3p, hsa-miR-24-3p, and hsa-miR-125b-5p in WM9, WM35, and WM902B [39]. This shows, the distribution and expression levels of miRNAs in melanoma-derived exosomes are different due to species and tumor progression. Clinically, different miRNA profiles were also observed in the first and second melanoma from the same patient, such as miR-92b-3p, miR-205-5p, miR-200b-5p, and miR-149-5p that are significantly upregulated in the second melanoma compared to the first one [40]. In general, the miRNA profile in melanoma-derived exosomes is distinguished from that in parent cells or other cells, and the majority of these miRNAs are implicated with the survival and growth of melanoma.

## 4. Roles of Exosome-Derived miRNAs in Melanoma Progression

Melanoma is a malignant transformation of melanocytes, and understanding the mechanisms underlying melanoma progression is essential for its treatment. Recently, numerous studies focusing on exosomes indicate their potential roles in melanoma progression [41], including invasion and metastasis, angiogenesis, the tumor microenvironment and immune escape of melanoma (as shown in Table 1), which are believed to be mostly dependent on the biologically active molecules, especially miRNA, carried in exosomes.

### 4.1. Exosome-Derived miRNAs in Melanoma Invasion and Metastasis

Melanoma is an aggressive cancer type, with a high proclivity for invasion and metastasis early in the disease process, in which exosome exhibits propulsive effects. For example, the metastatic activity of highly metastatic melanoma cells can be transferred to poorly metastatic ones through uptake of exosomes released from highly metastatic cells [42,43,44]. Additionally, reduced generation of pro-invasive exosomes due to loss of Rab 27a expression in melanoma cell lines prevented its invasion and cell motility in vitro, as well as spontaneous metastasis in vivo [57]. MiRNA, as the most essential and crucial regulator in exosomes, could affect melanoma cell invasion and metastasis by targeting melanoma cells or other cells such as fibroblasts. Cancer stem cells (CSCs) are a special subpopulation of cancer cells with the characteristics of self-renewability and the ability to initiate, supplement and expand human tumors [58]. They have been isolated from different human solid tumors, including melanoma. Communication between CSCs and cancer cells plays crucial roles in metastatic dissemination, which greatly depends on exosomal miRNAs. It was demonstrated that high metastatic melanoma stem cells (OL-SCs) can transfer miR-4535 and miR-1268a through exosomes into low metastatic melanoma cells (MPCs) and enhance the metastatic ability of the latter by targeting autophagy pathway [42,43]. Similarly, low metastatic melanoma cells (M14-OL) also acquire metastatic colonization capability from a high metastatic melanoma cell line (M14-POL) through intaking M14-POL-derived exosomal miR-411-5p, which can activate ERK signaling pathway [44]. Additionally, miR-222 was shown as a key factor for melanoma development and dissemination, not only because of its down-modulation of several direct targets, such as cyclin-dependent kinase inhibitor 1B (p27Kip1/CDKN1B), KIT proto-oncogene (c-KIT) receptor, and Fos proto-oncogene (c-FOS), but also due to its delivery through exosomes into surrounding low metastatic melanoma cells to improve their migration and invasion ability [41]. In addition, it was identified that P2X7 receptor (P2X7R) stimulation triggers melanoma cells to release microvesicles and exosomes, and also induces a change in the miRNA composition such as miR-495-3p, miR-376c-3p, and miR-6730-3p, which are strongly upregulated in all vesicles subtypes and contribute to melanoma growth and metastasis [45]. Among them, miR-495-3p was reported to target E2F transcription factor 3 (E2F3), which could induce melanoma cell proliferation, migration, and invasion by promoting the transformation of G1 to S [59,60,61]. Moreover, Kim et al. [46] found that exosomes from antigen-stimulated RBL2H3 cells increased the tumorigenic and metastatic potentials of B16F1 melanoma cells in a miR-154-5p-dependent manner. Interestingly, miR-2478 derived from milk exosomes can inhibit the expression of RAS-related protein 1a (RAP1A) in melanoma cells, thereby promoting the activation of protein kinase B (AKT) and inactivation of glycogen synthase kinase 3 beta (GSK3β), and ultimately leading to the inhibition of microphthalmia-associated transcription factor (MITF) expression [47]. Decreased expression of MITF was reported to promote the migration ability of melanoma cells [62].

**Figure 3:**
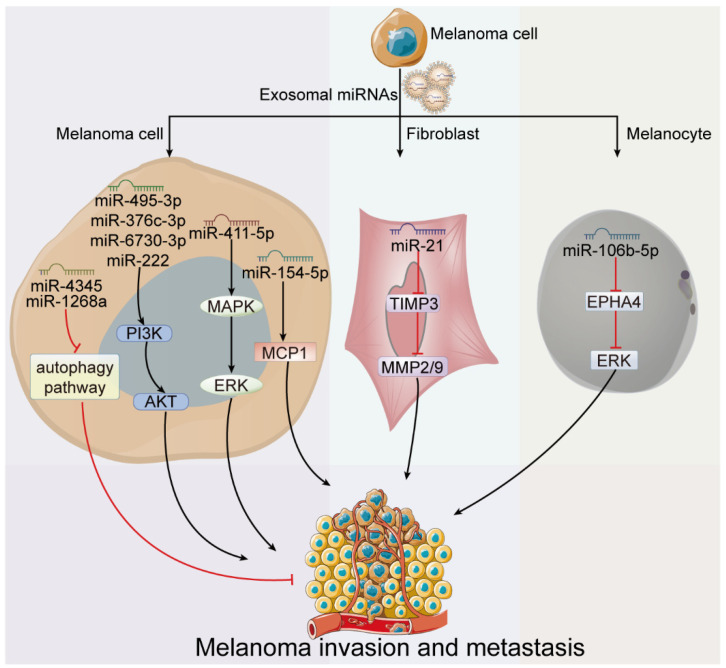
Roles of exosome-derived miRNAs in melanoma invasion and metastasis. Exosomes derived from high metastatic melanoma cells promote melanoma invasion and metastasis through delivering miRNAs into low metastatic melanoma cells to increase the invasive ability of the latter, or into the fibroblasts or the melanocytes to generate the pro-invasive surroundings.

Moreover, exosome-derived miRNAs could also work on other cells to facilitate melanoma invasion. It was reported that exosome-derived miR-21 from B16F10 cells can induce fibroblasts to be more invasive with down-regulation of tissue inhibitor of metalloproteinase 3 (TIMP3) expression and up-regulation of matrix metalloprotein (MMP) expression, which rearrange extracellular matrix (ECM) to facilitate melanoma cells migration and invasion [48]. Moreover, Luan et al. [49] found that exosomes derived from melanoma cells can shift miR-106b-5p to melanocytes, inducing epithelial mesenchymal transition (EMT) of melanocytes via EPHA4/ERK pathway to promote melanoma metastasis. Similarly, Xiao et al. [50] demonstrated that melanoma-derived exosomes promote phenotype switching in primary melanocytes through regulating MAPK pathway and let-7i. They also discovered that EMT-related miRNAs (miR-191and let-7a) in exosomes were significantly higher in melanoma patients compared to non-melanoma subjects. Moreover, miR-191 has been reported to promote Wnt/β-catenin pathway by suppressing brain abundant membrane attached signal protein 1 (BASP1) and upregulating WT1 transcription factor (WT1), thus having an impact on EMT [63]. Until now, many exosomal miRNAs that have been linked to or reported to be involved in the metastasis process of melanoma still remain understudied. It was shown that prevention of shifted exosome-shuttled miR-494 can suppress melanoma growth and metastasis, although the targeting cells are not defined [51]. Accordingly, exosome-derived miRNAs play an essential role in melanoma invasion and metastasis through altering capacity of melanoma or extracellular matrix around melanoma (as shown in Figure 3).

### 4.2. Exosome-Derived miRNAs in Melanoma Microenvironment

Melanoma is not an isolated entity but rather depends on the adjacent microenvironment, including fibroblasts, immune cells, blood vessels, and extracellular matrix (ECM). This microenvironment is an active promoter supporting melanoma survival, local invasion, and metastatic dissemination [64], and its establishment is inextricably linked to exosome-derived miRNAs that not only “educate” fibroblasts and macrophages to form CAFs and tumor associated macrophages (TAMs) to facilitate melanoma progression, but also disseminate through the circulatory system to induce formation of microenvironments in distant organs, termed as pre-metastatic niches (PMNs).

CAFs are a major component of the tumor stroma and various studies indicated that melanoma cell-secreted exosomes can induce the reprogramming of fibroblasts into CAFs [38,48,52]. For example, exosomes derived from B16F10 cells can be endocytosed by fibroblast cells, and induce the phenotypic shift of fibroblasts characterized by increased invasiveness. This process was mediated by miR-21 that is abundantly concentrated in melanoma exosomes and frequently overexpressed in numerous malignancies [48]. Moreover, exosomal miR-155 from B16F10 cells was also demonstrated to trigger the proangiogenic switch of CAFs by directly targeting suppressor of cytokine signaling 1 (SOCS1). Downregulation of SOCS1 further activates Janus kinase 2/signal transducer and activator of transcription 3 (JAK2/STAT3) signaling pathway and elevates the expression levels of vascular endothelial growth factor A (VEGF-A), fibroblast growth factor 2 (FGF2), and MMP9 in fibroblasts, which eventually contributes to angiogenesis [38]. Melanosome, as one of extracellular microvesicles carrying miRNAs, can be untaken by primary fibroblasts and reprogram fibroblasts into CAFs, manifested as increased proliferation and cell motility, enhancement in collagen contraction and cytokines production such as interleukin 1 beta (IL-1β), interleukin 6 (IL-6), and interleukin 8 (IL-8) [52]. And this fibroblast reprogramming is dependent of melanosome derived miR-211, which is delivered into fibroblasts and directly targets insulin growth factor 2 receptor (IGF2R) leading to mitogen-activated protein kinase (MAPK) signaling activation, and reciprocally encourages melanoma growth. Notably, miR-211 was also reported to exist in melanoma exosomes [65], and the miR-211 level was observed higher in melanoma metastases than primary melanoma tissues [66], thus it follows naturally that exosomes, as a communication vehicle, might likewise transfer miR-211 to reprogram fibroblasts to CAFs. Except for melanoma primary niche formation, melanoma-derived exosomes could also be captured by normal stromal fibroblasts in distal sites through the lymphatic system and blood vessels. These distal sites are gradually preconditioned into pre-metastatic sites due to the metabolic reprogramming of fibroblasts mediated by miR-155 and miR-210 delivered from melanoma exosomes, leading to increased acidity of the microenvironment, which eventually contributes to the development of melanoma metastasis [53].

Tumor-associated macrophages (TAMs), the most abundant immune cells in tumor microenvironment, are not typically M1 or M2 macrophage phenotype. Rather, TAMs often express high amounts of pro-inflammatory, proangiogenic, and chemotaxis-inducing cytokines and chemokines and then facilitate tumor growth, progression, metastasis, angiogenesis, and immune evasion [67,68,69]. Numerous studies reported that exosomes, especially exosome-derived miRNAs, induce a tumor-promoting phenotype in macrophages. Wang et al. [70] showed that melanoma-derived conditioned media efficiently induce the differentiation of monocytes to macrophages with upregulation of genes mainly associated with tumor invasion. Similarly, Bardi et al. [71] further reported that melanoma exosomes can induce a mixed M1 and M2 pro-tumor activation phenotype with elevation of tumor necrosis factor (TNF-α), IL-1β, interleukin 10 (IL-10), and transforming growth factor beta 1 (TGF-β). Mechanically, Gerloff et al. [39] identified that this macrophage phenotype switch is attributed to exosome-delivered miR-125b-5p, which is delivered from melanoma cells into THP-1-derived macrophages and directly targets the lysosomal acid lipase A (LIPA). As known, LIPA is a crucial regulator for fatty-acid oxidation, which is significant for the polarization of anti-inflammatory M2 macrophages [72]. Until now, studies on the mechanism of melanoma exosome reprogramming TAMs are limited; however, it is significant to refer to other tumors. It was demonstrated that exosomal miR-21 derived from bladder cancer cells could suppress phosphatase and tensin homolog (PTEN) of phosphatidylinositol-4,5-bisphosphate 3-kinase catalytic subunit alpha/AKT (PI3K/AKT) signaling pathway and activate STAT3 expression to promote M2 phenotype polarization in macrophages [73], and miR-34a from triple negative breast cancer can promote M2 macrophage polarization through MCTS1 re-initiation and release factor (MCT-1)/miR-34a/IL-6/IL-6R signal axis [74]. Notably, miR-21 and miR-34a are identified to exist abundantly in melanoma exosomes [75], which indirectly indicates the potential of melanoma exosomes-derived miRNAs in TAMs induction.

**Figure 4:**
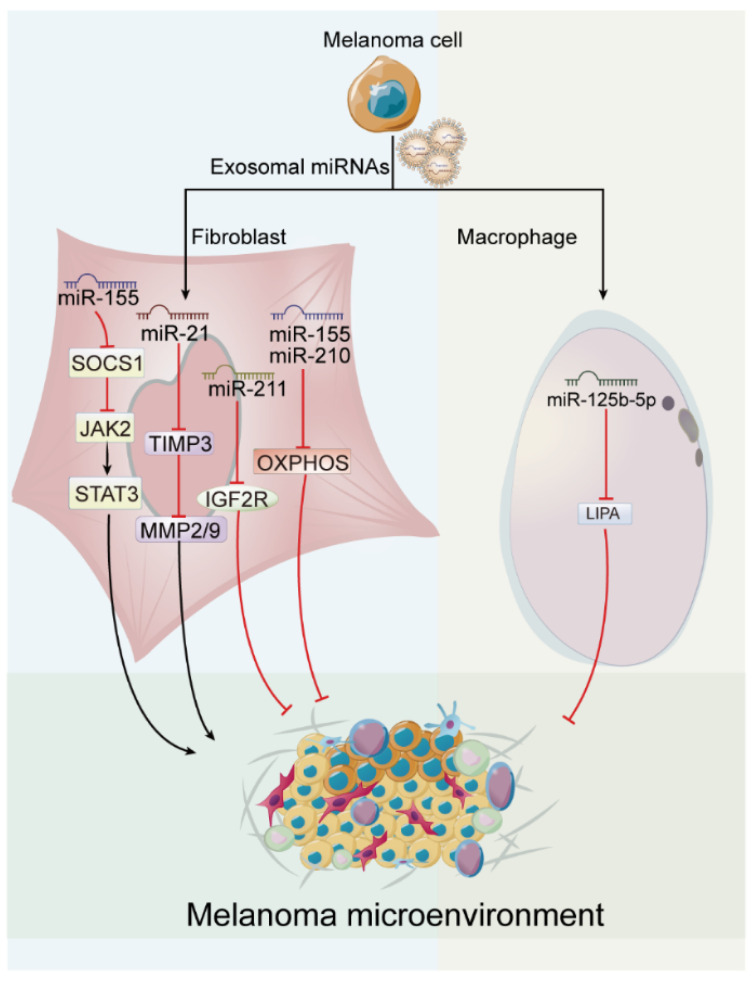
Roles of exosome-derived miRNAs in melanoma microenvironment. Exosomal miRNAs generate a melanoma-friendly microenvironment through inducing fibroblasts and macrophages into cancer-associated fibroblasts (CAFs) and tumor-associated macrophages (TAMs), respectively.

Collectively, exosome-derived miRNAs are crucial in the establishment of melanoma microenvironment or pre-metastatic niches (as shown in Figure 4).

### 4.3. Exosome-Derived miRNAs in Melanoma Angiogenesis

**Figure 5:**
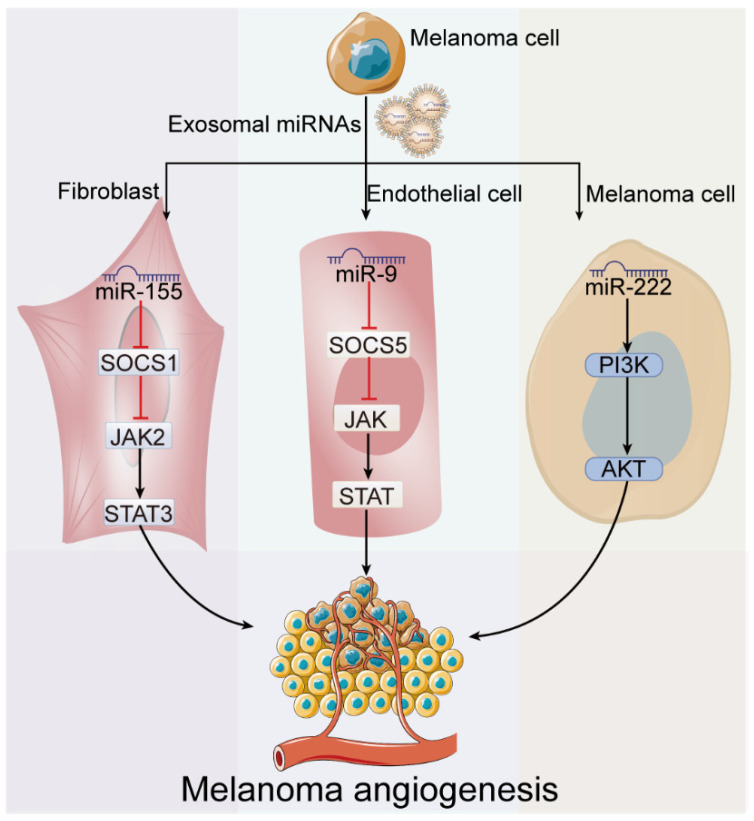
Roles of exosome-derived miRNAs in melanoma angiogenesis. On the one side, exosome-derived miRNAs regulate melanoma microenvironment to facilitate angiogenesis through working on fibroblasts or melanoma cells. On the other sides, exosomal miRNAs from melanoma can be uptaken directly by endothelial cells to induce angiogenesis.

Given the hypoxia caused by fast proliferation of tumor cells, angiogenesis is essential for melanoma progression. Except for hypoxia-inducible factor 1 subunit alpha (HIF-1α) induced by hypoxia condition that regulates its downstream VEGF-A directly, exosome-derived miRNAs also play essential roles in this process. On the one side, melanoma exosomes regulate TME to facilitate angiogenesis. For example, melanoma exosomal miR-155 targets SOCS1 in fibroblasts to stimulate pro-angiogenic factors, such as VEGF-A, FGF2, and proteolytic enzymes [38]. Melanoma exosomes also induce endothelial cell GM-CSF production in pre-metastatic lymph nodes, which results in different M1 and M2 macrophage-mediated angiogenic processes [76]. On the other side, exosomal miRNAs from melanoma can be up-taken directly by endothelial cells to induce angiogenesis. MiR-9, transferred to endothelial cells from melanoma through exosomes, was demonstrated to effectively reduce SOCS5 levels, leading to activated JAK-STAT pathway, which promotes endothelial cell migration and angiogenesis [54]. However, miR-222, as the main miRNA in melanoma exosomes, shows an opposite role on angiogenesis when delivered to endothelial cells (EC), in spite of its pro-angiogenesis effect in melanoma [77]. Generally, exosome-derived miRNAs contribute significantly to angiogenesis, but the specific roles and mechanisms still need further exploration (as shown in Figure 5).

### 4.4. Exosome-Derived miRNAs in Melanoma Immune Escape

**Figure 6:**
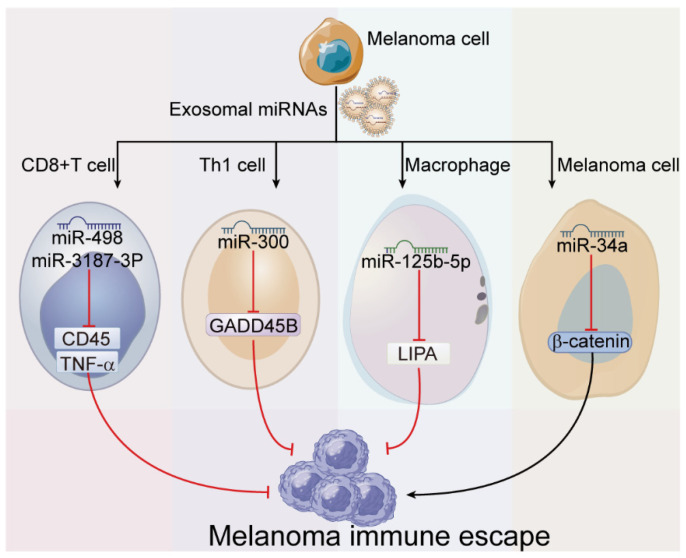
Roles of exosome-derived miRNAs in melanoma immune escape. MiRNAs derived from melanoma exosomes could suppress the abilities of CD8^+^ T cells and Th1 cells to target and attack melanoma cells, encourage the development of tumor-associated macrophages, and reduce the antitumor immunity of melanoma, all of which ultimately contribute to immune escape.

Melanoma is one of the most immunogenic tumors, and evading immune system is a necessary step for its survival and metastases. The immune escape ability of melanoma is partially gained from exosome-derived miRNAs. Vignard et al. [36] showed that melanoma-derived exosomes could be internalized by immune cells, such as monocytes, T, and NK cells, but not B cells. Among them, CD8^+^ T cells lose their ability to induce the death of melanoma cells leading to immune escape after exposure to melanoma-derived exosomes, manifested as reduced TNF-α secretion and damaged T-cell receptor (TCR) signaling pathway. Further study indicated that reduction of TNF-α is associated with hsa-miR-181 family, hsa-miR-122, hsa-miR-149, hsa-miR-498, and hsa-miR-3187-3p enriched in melanoma-derived exosomes, especially hsa-miR-181 family and hsa-miR-498 interact directly with the 3′-UTR sequence of TNF-α. Moreover, CD45, a crucial regulator for successful antigen receptor signaling in T cells [78], was also downregulated on CD8^+^ T cells after uptake of melanoma-derived exosomes, which is mediated by carried hsa-miR-3187-3p through directly targeting protein tyrosine phosphatase receptor type C (PTPRC) 3′-UTR. Similarly, melanoma was also reported to export miR-300 via exosomes [55], and miR-300 can bind to growth arrest and DNA damage inducible beta (GADD45B) 3′-UTR to inhibit its expression that is usually highly up-regulated during Th1 type responses against cancer cells [79]. GADD45B is crucial in the initiation of Th1 type response through controlling the production of interferon-γ (IFN-γ), and down-regulation of GADD45B weakens the activity of p38 MAP kinase, leading to the reduction of IFN-γ. In addition, the deletion of GADD45B also decreases the mRNA of T-bet and Eomes, which further supports the critical role of GADD45B in shaping the Th1 fate [79,80]. Therefore, exosome-derived miR-300 is implicated in melanoma immune escape, and miR-300 was identified to clinically linked to worse prognosis in melanoma patients [55]. In addition, melanoma exosome-delivered miR-125b-5p contributes to the phenotypic transformation of macrophages with the induction of C-C motif chemokine ligand 1(CCL1), CCL2, and IL-1β expression, thus leading to myeloid cell recruitment and cancer-associated inflammation [39]. Furthermore, the CD80 expression induced by miR-125b was shown to have an increased binding affinity to the immune checkpoint molecule cytotoxic T-lymphocyte associated protein 4 (CTLA4) on activated T cells, which ultimately inhibits the proliferation and function of activated T cell [81]. In turn, exosomal miRNAs have also been shown to work on melanoma cells to affect the immune surveillance. Lee et al. [56] identified that miR-34a in innate extracellular vesicles from melanoma patients could suppress the expression of β-catenin in melanoma cells. As known, β-catenin pathway plays an important role in the antitumor immunity of melanoma [82]. Taken together, exosomal miRNAs make significant contributions to the interaction between the immune system and melanoma cells (as shown in Figure 6).

## 5. Exosome-Derived miRNAs as Potential Marker for Melanoma Diagnosis

Nowadays, miRNAs are regarded as one of the novel epigenetic markers for diagnosis and/or prognosis in many diseases, including melanoma. Hundreds of miRNAs were identified as potential diagnostic and prognostic tools in melanoma, which are usually dysregulated in melanoma and associated with melanoma progression [66]. Rosenfeld et al. [83] constructed a classifier composed of 48 miRNAs based on miRNA microarray data of 253 samples from 22 different tumor tissues and metastases, which can identify the tissue origin of metastatic tumors. Nevertheless, higher accuracy requires more miRNAs, and it is also challenging to distinguish small sets of informative genes from noise or natural variation due to the small sample sizes available. Moreover, the majority of these miRNAs were detected in tissue samples, and the inaccessibility of samples limits its further application. Exosomes, abundantly secreted by melanoma cells, can exist stably in blood, urine, and other body fluids and their bilayer membrane structure makes the miRNAs carried more stable. Moreover, exosomes are the principal carriers of circulating miRNAs, albeit there are other ones as well, such as the high-density lipoproteins or the Argonaute 2 protein. Thus, circulating miRNAs, especially exosome-derived miRNAs, are advantageous and feasible as melanoma biomarkers due to less invasiveness and more convenience (as summarized in Table 2).

Owing to the characteristic miRNAs and proteomic profiles in melanoma-derived exosomes comparing to that from normal melanocytes, the miRNA levels in these vesicles may serve as an effective biomarker for malignant melanoma. Evidence showed that the circulating miR-17, miR-19a, miR-21, miR-126, and miR-149 in plasma-derived exosomes from patients with metastatic sporadic melanoma were significantly higher than that from patients with familial melanoma or unaffected control subjects [92]. Similarly, Ragusa et al. [75] identified miR-21, miR-34a, and miR-146a were up-regulated in vitreal exosomes from uveal melanoma patients with respect to healthy controls. Interestingly, miR-146a was also upregulated in the serum of uveal melanoma patients, as well as in serum exosomes. Moreover, there is significant upregulation of miR-191 and let-7a in serum exosomes of stage I melanoma patients as compared with the non-melanoma subjects [50]. Additionally, miR-532-5p and miR-106b detected in serous exosomes differed between patients with melanoma and healthy individuals. Not only that, these exosomal miRNA panels also distinguished patients with metastasis from those without metastasis, and patients at stage I-II from those at stage III-IV [93]. Instead, Alegre et al. [94] demonstrated a significant reduction of exosomal miR-125b levels in melanoma patients compared with disease-free melanoma patients and healthy controls. Lower levels of miR-125b in exosomes obtained from serum are associated with advanced melanoma disease, probably reflecting the tumoral cell dysregulation. Intriguingly, most of these potential miRNA markers have been shown to be essential for melanoma progression in preclinical studies. For example, reduced exosomal miR-1180-3p in melanoma patient plasma is indicated as a promising diagnostic marker of melanoma, and the level of miR-1180-3p was also verified lower in melanoma cell line than that in melanocytes and negatively associated with melanoma proliferation, migration, and invasion by targeting ST3 beta-galactoside alpha-2,3-sialyltransferase 4 (ST3GAL4) [96].

The changes in these miRNAs in serum exosomes indicate their potential roles as diagnostic markers for melanoma. However, small clinical sample sizes and less refined classification limit these miRNAs as reliable and accurate indicators for melanoma diagnosis cross-patient and cross-cohort. Moreover, some miRNAs are common, and highly expressed in many other cancers except for melanoma, such as miR-21 that is detected in colon cancer [101], hepatocellular carcinoma [102], oral cancer [103], breast cancer [104] and so on, and also positively correlated with their progression. This suggests that it is challenging to distinguish melanoma from other cancers by using a single specific miRNA. Therefore, large-scale clinical samples, detailed classification, and determination of more specific miRNAs will aid in the discovery of unique markers. Interestingly, Wozniak et al. [95] discovered that the expression of miR-494-5p, miR-4497, and miR-513a-5p in melanoma exosomes are strongly up-regulated, while miR-125b-5p and miR-3934-5p decrease under hypoxia condition. Considering that hypoxia is a prominent feature in the microenvironment of melanoma and is associated with melanoma growth and progression, these miRNAs related with hypoxia will be an alternative for melanoma diagnosis. In addition, exosomal miRNAs can also be used to predict therapeutic responses. MAPK pathway inhibitors (MAPKis), a novel targeted therapy for BRAFV600 mutant cutaneous malignant melanoma, are receiving great attention, which induce rapid responses and improve treatment outcome. However, the efficacy is limited due to emerging resistance. It was revealed that elevated extracellular microvesicle let-7g-5p is connected with improved disease control, while increased extracellular microvesicle miR-497-5p is associated with longer progression free survival during the treatment of MAPKis. Therefore, let-7g-5p and miR-497-5p were identified as novel predictive biomarkers for the presumed therapeutic benefit of MAPKi in patients with cutaneous malignant melanoma [90]. Overall, exosomal miRNAs receive more and more attention as non-invasive biomarkers to indicate disease status [105].

## 6. miRNAs Based on Exosomes in Melanoma Treatment

Until now, surgical resection, chemotherapeutic agents and radiation therapy are the principal choices for melanoma treatment. However, strong side effects and high recurrence rate lead us to seek more efficient and precise, and safer treatments [106,107,108]. Although new targeted therapies such as vemurafenib and dabrafenib, that are the B-Raf proto-oncogene (BRAF) inhibitors, have shown initial promising anti-tumor responses in melanoma patients, drug resistance continues to be a major barrier for melanoma therapy [109,110,111]. The mechanism of drug resistance has not yet been clearly elucidated, but the secretion of tumor-suppressive miRNA through exosomes was speculated to be involved. Ge et al. [112] found that the treatment of cisplatin, a traditional chemotherapeutic drug, promotes the exosomal secretion of miR-34a from melanoma A375 cells, resulting in the resistance to cisplatin-induced apoptosis. Moreover, miR-222, miR-488-3p, miR-195, and miR-211 were also reported to participate in the resistance to modalities of ipilimumab and cisplatin [66]. Given the importance of exosome-derived miRNAs in melanoma progression, targeting miRNAs and exosomes becomes a potential therapeutic strategy, receiving considerable attention.

So far, many new miRNA-based cancer therapies have been developed, and there are two strategies for cancer therapy known as replacement or overexpression therapy as miRNA mimics and inhibition or downregulation therapy as chemically modified antisense oligonucleotides (antagomiRs). Locked nucleic acid (LNA), one of the most common varieties of antisense oligonucleotides, has a therapeutic potential with low toxicity, elevated affinity, and high stability and specificity in vivo [113]. Javanmard et al. [114] demonstrated that suppressing miR-21 through LNA-anti-miR-21 can effectively decrease the proliferation and increase the apoptosis of mouse melanoma cells (B16F10) and also reduce tumor growth and volume in the model of melanoma in male C57BL/6 mice grafted with B16F10 cells, suggesting LNA-anti-miR-21 as a potential therapeutic option for patients with melanoma. Moreover, miR-1908, miR-199a-5p, and miR-199a-3p were identified as endogenous promoters of metastasis in melanoma, which convergently target apolipoprotein E signaling, leading to invasion and endothelial recruitment through LDL receptor-related protein 1/8 (LRP1/LRP8) receptors. Therefore, LNA-anti-miR-199a-3p, LNA-anti-miR-199a-5p, and LNA-anti-miR-1908 were used to inhibit metastatic activity of highly metastatic MeWo-LM2 cells, showing great therapeutic potential in a variety of human melanoma strains with BRAF and NRAS proto-oncogene (NRAS) mutation states [115]. On the other side, tumor suppressor miRNAs also achieve therapeutic purposes through replacement or mimic introduction. For miRNA replacement therapy, restoration of miR-126-3p inhibited expression of VEGF-A and ADAM metallopeptidase domain 9 (ADAM9) and activation of ERK1/2 and/or AKT, suppressed proliferation, invasiveness, and finally improved the drug sensitivity in dabrafenib-resistant melanoma [116]. Moreover, Zheng et al. [117] revealed that ectopic expression of miR-224-5p inhibited cell proliferation, cell cycle, and migration in uveal melanoma by directly targeting Rac family small GTPase 1 (RAC1) and FIZZLED 5. MRX34, a liposomal miR-34a mimic that can downregulate over 30 oncogenes across multiple oncogenic pathways, has been assessed clinically in phase I study. And treatment of MRX34 combining with dexamethasone premedication exhibited antitumor activity and acceptable safety in patients with refractory advanced solid tumors, which demonstrated the clinical potential of miRNAs as an effective therapy for malignancies including melanoma [118]. In addition, some natural anti-cancer drugs have been found to target miRNA for melanoma treatment. For example, curcumin and its analogues could inhibit the migration and invasion of melanoma cells and induce their apoptosis [119,120], which is partially through regulating miRNAs [121], such as miR-21/PTEN/PDCD4 axis [122]. Tanshinol also shows antitumor effect on melanoma by regulating miR-1207-5p/chondroitin polymerizing factor (CHPF) [123]. Genistein, the isoflavone extracted from soybean, has been shown to suppress proliferation of human uveal melanoma cells possibly through modulating expression of miR-27a and its target gene zinc finger and BTB domain containing 10 (ZBTB10) [124]. It is important to note that the variety of downstream regulation of miRNAs introduces uncertainties in its application for therapy. Nevertheless, several miRNAs are presently being tested in clinical trials of some diseases. For instance, miR-122/miravirsen and miR-92/MRG-110 produced by Roche/Santaris and Regulus Therapeutics, respectively, might be the pioneer products for melanoma medicine development [125].

Owing to the high membrane permeability and low cytotoxicity as well as increased stability and biocompatibility, exosomes have already become an important option for drug delivery. For example, many drugs cannot cross the blood–brain barrier, while the drugs encapsulated in endothelial cell-derived exosomes effectively penetrated this barrier [3]. In clinical trials, exosomes loaded with MAGE3 derived from dendritic cells were injected intradermally to stimulate the immune response in melanoma patients of stage IIIb/IV. In the phase I trial, 27% of metastatic melanoma patients derived good response [126]. Furthermore, it will be a promising therapeutic strategy to employ the exosomes as delivery vehicles carrying natural or synthetic miRNAs or mimics or antagomiRs. According to the target miRNA, appropriate exosome-producing cells should be selected. The key aspect of exosomes selection is the scalability and production efficiency of exosomes, and the standardization of exosomes separation, quantification, and characterization [127]. Before the exosomes are used for treatment, some steps are required for characterization, including total exosome count, protein count, lipid count, RNA count, protein maker, lipid maker, structure, size, chemical composition, and topology [128]. It was reported that miR-503 enriched exosomes isolated from endothelial cells successfully impaired tumor cell proliferation and invasion in vitro [129], and miR-134 encapsulated by exosome reduced migration and invasion ability of breast cancer cells [130]. Moreover, there are also other ways to introduce target miRNAs into exosomes artificially as needed, such as electroporation [131], exosome transfection reagent [132], co-incubation [133], etc. Among them, electroporation is attracting interest because of its high loading efficiency. However, the impact on the integrity of EVs and EVs aggregation risk limits the use of electroporation. In addition, electroporation may affect the zeta potential and colloidal stability of EVs, leading to aggregation of RNA and EVs [134]. To solve this problem, Hood et al. [135] developed a new trehalose pulse culture medium, which can improve the colloidal stability of EVs and reduce the aggregation caused by electroporation. The combination of miRNAs based on exosomes with traditional treatment dramatically improves the efficacy. Recent studies have shown that engineered exosomes can encapsulate miRNAs and chemotherapeutic drugs at the same time, realizing enhanced anticancer effects [136]. More importantly, engineering exosomes, that embed homing peptides or specific antibodies on their surface through glycosylation, click chemistry or non-covalent modification, can target specific cells, so that targeted therapy of melanoma will be possible. However, many synthetic peptides designed on the surface of exosomes might be degraded during exosome biogenesis by cellular enzymes, and glycosylation of signal peptides provides an effective method to reduce the degradation and improve stability. For example, Hung et al. [137] demonstrated that peptides fused to the N-terminus of exosome-associated transmembrane protein lysosomal-associated membrane protein 2b (LAMP2B) were cleaved in samples from both cells and exosomes, and introduction of glycosylation motif prevented the peptide from degrading. Notably, the glycosylated peptides not only retain the ability to bind homologous peptide binding proteins, but also protect the targeted peptides to enhance the uptake of exosomes by receptor cells. To sum up, targeting miRNA based on exosomes is a potential therapeutic strategy for melanoma treatment.

## 7. Future Perspectives and Conclusion Remarks

In general, exosome-derived miRNAs play critical roles in mediating melanoma progression including melanoma metastasis, microenvironment establishment, angiogenesis, and immune escape, and exhibit the great potential to be markers for melanoma diagnosis and therapy target. However, the mechanisms behind them need to be further delineated. On the one side, there are numerous studies on the function of exosomes in melanoma, but the detailed mechanism and involved miRNAs still need to be clarified. As a result, many functional miRNAs originating from melanoma exosomes are undefined. Moreover, the database linking exosomal miRNAs to pathogenesis, disease course, classification and prognosis of melanoma is insufficient, and it is also tough to identify melanoma from other cancers due to the universality and non-specificity of miRNA, which are challenges for exosomal miRNAs as melanoma diagnostic markers. Thus, more clinical information on the miRNAs produced from melanoma exosomes is required in the future, and then melanoma prediction models or system should be developed, based on artificial intelligence (AI) through in-depth study of clinical data, including cancer type, miRNA expression level, and patient information, so as to evaluate the best miRNA candidates or targets. On the other side, the complex regulatory network of miRNA makes the therapy based on miRNA untargeted. According to the analysis of the entire miRNA-mRNA reconstruction network of prostate cancer [138], each individual miRNA has an average of 51 target genes, and each gene has four co-regulatory miRNAs. Accordingly, it is critical to solve the question of how to focus on targeted pathways and avoid alternative risk pathways.

Although there are still challenges for exosomal miRNAs in melanoma diagnosis and effective therapy, many efforts and attempts are still encouraging. Some natural anticancer agents attract great attention and bring new ideas because of their targeting on miRNA regulation. Moreover, the construction of engineered exosomes as transporting vehicles may provide a safe, efficient, and targeted therapy for melanoma.

## Figures and Tables

**Table 1:** Exosome-derived miRNAs in melanoma.

Foundation	miRNA	EP	Target Cell	Target Gene	Mechanism	Ref
Invasion and metastasis	miR-4345	-	Low metastatic melanoma cell	-	autophagy pathway	[42]
	miR-1268a	-	Low metastatic melanoma cell	-	autophagy pathway	[43]
	miR-411-5p	-	Low metastatic melanoma cell	-	MAPK/ERK pathway	[44]
	miR-222	-	Melanoma cell	p27Kip1/CDKN1B, c-KIT receptor and c-FOS	PI3K/AKT pathway	[41]
	miR-495-3p miR-376c-3p miR-6730-3p	-	Melanoma cell	-	PI3K/AKT pathway	[45]
	miR-154-5p	-	Melanoma cell	MCP1	-	[46]
	miR-2478	-	Melanoma cell	RAP1A	Akt/GSK3β pathway	[47]
	miR-21	↑	Fibroblast	TIMP3 and MMP	-	[48]
	miR-106b-5p	↑	Melanocyte	EPHA4	EPHA4/ERK pathway	[49]
	miR-191 let-7i let-7a	↑	Primary melanocyte	LIN28B and HMGA2	EMT	[50]
	miR-494	↓	-	Bcl-2	-	[51]
Tumor microeniron-ment	miR-21	↑	Fibroblast	TIMP3 and MMP	-	[48]
	miR-155	↑	CAF	SOCS1	JAK2/STAT3 pathway	[38]
	miR-211	↑	Fibroblast	IGF2R	MAPK pathway	[52]
	miR-155 miR-210	↑	Stromal fibroblast	-	OXPHOS	[53]
	miR-125b-5p	↑	Macrophage	LIPA	switch of TAM phenotype	[39]
Angiogenes-is	miR-155	↑	Fibroblast	SOCS1	JAK2/STAT3 pathway	[38]
	miR-9	↑	Endothelial cell	SOCS5	JAK/STAT pathway	[54]
	miR-222	-	Melanoma cell	-	PI3K/AKT pathway	[41]
Immune escape	miR-122 miR-149 miR-498 miR-181 miR-3187-3p	-	CD8^+^ T cell	TNF-α PTPRC	-	[36]
	miR-300	↓	Th 1 cell	GADD45B	-	[55]
	miR-125b-5p	↑	Macrophage	LIPA	switch of TAM phenotype	[39]
	miR-34a	-	Melanoma cell	β-catenin	-	[56]

EP, expression pattern in melanoma; Ref, references; ↑, upregulated; ↓, downregulated; -, unknown.

**Table 2:** Circulating miRNAs as potential markers for melanoma diagnosis.

miRNA	EP	Samples	Target	Effect	Refs
miR-211-5p	↑	Serum	-	Associated with disease stage and survival	[84]
miR-16	↑	Serum	-	Associated with disease stage	[84]
miR-4487	↓	Serum	-	Associated with diseases stage and survival	[84]
miR-221	↑	Serum	-	Associated with patient survival and advanced clinical stage	[85]
miR-23a	↓	Serum	ATG12	Associated with patient survival and tumor thickness	[86]
miR-150-5p	↓	Serum	-	Associated with patient survival	[87]
miR-142-3p	↓	Serum	-	Distinguish between stage III and stage IV	[87]
miR-206	↓	Serum	-	Associated with poor prognosis and clinical stage	[88]
miR-106b-5p	↑	Serum exosome	EPHA4	Activates the ERK pathway	[49]
miR-138	↓	Whole blood	-	Associated with survival	[89]
let-7g-5p	↓	Plasma exosome	MAPK	High expression levels associated with better disease control	[90]
miR-34a	↑	Plasma exosome	β-catenin	Prevents tumor relapse and suppresses tumor cell proliferation	[56]
miR-146a miR-155 miR-125b miR-100 miR-125a miR-146b miR-99b	↑	Melanoma exosome	CTLA4 PD-1	Convert myeloid cells into myeloid-derived suppressor cells	[91]
miR-495-3p miR-376c-3p miR-6730-3p	↑	Melanoma exosome	-	Promote melanoma growth and metastasis	[45]
miR-191 let-7a	↑	Serum exosome	-	Regulate the EMT process	[50]
miR-122 miR-149 miR-498 miR-3187-3p	↑	Melanoma exosome	TNF-α PTPRC	Promote melanoma immune escape	[36]
miR-17 miR-19a miR-21 miR-126 miR-149	↑	Plasma exosome	-	Associated with melanoma development and metastasis	[92]
miR-532-5p miR-106b	↑	Serum exosome	-	Associated with metastasis and disease stage	[93]
miR-125b	↓	Serum exosome	-	Associated with advanced melanoma	[94]
miR-494-5p miR-4497 miR-513a-5p	↑	Melanoma exosome	-	May be related to melanoma growth and progression	[95]
miR-125b-5P miR-3934-5p	↓	Melanoma exosome	-	May be related to melanoma growth and progression	[95]
miR-1180-3p	↓	Plasma exosome	-	Promote melanoma growth	[96]
miR-10b	↑	Serum	-	High levels associated with short disease-free survival and overall survival	[97]
miR-149-3p miR-150-5p miR-193a-3p	↑ ↑ ↓	Plasma	-	Triple classifier is suitable for early diagnosis of melanoma	[98]
miR-1246 miR-185	↑	Plasma		Distinguish metastatic melanoma	[99]
miR-9	↑	Serum		Distinguish metastatic melanoma	[100]

EP, expression pattern in melanoma; Refs, references; ↑, upregulated; ↓, downregulated; -, unknown.

## Abbreviations

miRNA/miR, microRNA; MVBs, multivesicular bodies; ILVs, intraluminal vesicles; ESCRT, endosomal sorting complex required for transport; EVs, extracellular vesicles; miRISC, miRNA induced silencing complex; ERK, extracellular signal regulated kinases; AKT, protein kinase B; PI3K, phosphatidylinositol-4,5-bisphosphate 3-kinase catalytic subunit alpha EMT, epithelial mesenchymal transition; CAFs, cancer associated fibroblasts; TAMs, tumor associated macrophages; SOCS, suppressor of cytokine signaling; VEGF-A, vascular endothelial growth factor A; TNF-α,tumor necrosis factor; IL-1β, interleukin 1 beta; LNA, Locked nucleic acid; CSC, cancer stem cell; MSC, melanoma stem cell.
